# Editorial: Efficacy of probiotic-enriched foods on digestive health and overall well-being

**DOI:** 10.3389/fnut.2026.1781349

**Published:** 2026-01-27

**Authors:** Silvia Amalia Nemes, Laura Mitrea, Lavinia Florina Calinoiu, Bernadette Emoke Teleky, Leontina Lipan, Dan Cristian Vodnar

**Affiliations:** 1 University of Agricultural Sciences and Veterinary Medicine of Cluj-Napoca, Cluj-Napoca, Romania; 2 Miguel Hernández University of Elche, Elche, Spain

**Keywords:** digestive health, functional food, gut microbiota, nutrition science, probiotics

The growing recognition of the gut as a central regulator of human health has driven intense scientific interest in probiotic-enriched foods. Advances in microbiome research have revealed the complex interactions between dietary components, gut microbials, and host physiological functions, positioning probiotics as promising modulators of digestive health and overall wellbeing (1, 2). Digestive health is fundamental to human wellbeing, influencing nutrient absorption, immune competence, metabolic regulation, and neuropsychological outcomes. Disruptions to gut microbial balance, often referred to as dysbiosis, have been associated with a range of gastrointestinal disorders, including irritable bowel syndrome, inflammatory bowel disease, and functional constipation, as well as systemic conditions such as obesity, metabolic syndrome, allergies, and mood disorders (3) (Zhang et al.; Chen et al.). Consequently, dietary interventions aimed at restoring or maintaining microbial balance have become a central point of preventive and therapeutic nutrition research. Unlike pharmaceutical preparations, probiotic foods enhance adherence and long-term applicability. However, evidence accumulated in recent years has emphasized that probiotic efficacy is not universal and depends on multiple factors, including microbial strain characteristics, dosage, food matrix, duration of intake, and host-specific variables (4). Research has demonstrated that selected probiotic strains can improve gastrointestinal function. Reported benefits include enhanced bowel regularity, attenuation of bloating and abdominal discomfort, and support of intestinal barrier integrity. These effects are frequently attributed to mechanisms including competitive inhibition of pathogenic microorganisms, modulation of gut motility, production of bioactive metabolites, such as short-chain fatty acids, and regulation of mucosal immune responses (5).

The role of the food matrix has also emerged as a critical determinant of probiotic functionality. Fermented foods, dairy products, and plant-based alternatives provide distinct physicochemical environments that influence microbial viability during processing, storage, and gastrointestinal transit. Research indicates that appropriately designed food carriers can enhance probiotic survival and metabolic activity, thereby increasing the physiological impact (4, 6). These findings highlight the importance of integrating microbiological and food technological considerations when developing probiotic-enriched products. Beyond localized digestive effects, growing evidence supports the influence of probiotic-enriched foods on systemic health outcomes. The gut-immune-brain axis has been extensively investigated, with studies indicating that probiotics modulate immune cell activity, cytokine profiles, and inflammatory markers. Such effects are particularly relevant in populations exposed to chronic low-grade inflammation, including older adults and individuals with metabolic or immune-related vulnerabilities (7). Incorporating probiotics into commonly consumed foods offers a practical approach to supporting immune resilience through everyday nutrition. Despite relevant findings, the evidence base also reveals important challenges that must be addressed to advance the field. Variability in study design, probiotic formulations, outcome measures, and participant characteristics complicates cross-study comparisons and limits the ability to draw universal conclusions. Differences in baseline microbiota composition, dietary habits, age, and health status further contribute to heterogeneous responses. These observations reinforce the necessity for well-designed human studies employing standardized methodologies and clearly defined endpoints.

In reaction to this heterogeneity, the concept of personalized probiotic strategy has gained popularity. Rather than assuming uniform responses to probiotic-enriched foods, current research increasingly acknowledges individual differences in microbial ecosystems (8). Consumer interest in functional foods continues to grow, driven by increased awareness of the links between diet and health. At the same time, the proliferation of probiotic products on the market raises concerns regarding the validation of health claims, strain transparency, and regulatory oversight. Strong scientific evidence remains essential to ensure that probiotic-enriched foods deliver benefits and maintain consumer trust. Interdisciplinary collaboration is therefore central to progress in this area. Advances require coordinated efforts across microbiology, nutrition, food science, clinical research, and regulatory frameworks. Addressing issues such as microbial stability, sensory acceptance, dose optimization, and long-term safety is crucial for translating laboratory findings into effective dietary solutions. Moreover, clear communication of evidence-based benefits and limitations is necessary to guide informed consumer choices.

In conclusion, probiotic-enriched foods represent a promising and evolving component of contemporary nutrition science. Accumulating evidence supports their potential to promote digestive health and contribute to broader aspects of wellbeing through interactions with gut microbiota, immune function, and metabolic and neurophysiological pathways. Future research should prioritize human studies, improved strain-level characterization, and integration of multi-omics approaches to deepen understanding of probiotic mechanisms and individual responsiveness. Strengthening the evidence base will facilitate the responsible development of probiotic-enriched foods and their effective incorporation into sustainable, health-promoting dietary patterns.
